# Case Records in a Teaching Hospital
**Clinical Records in the University of California Hospital*. Reprinted from the *Boston Medical and Surgical Journal*, November 1915.


**Published:** 1918-09-21

**Authors:** 


### CASE RECORDS IN A TEACHING HOSPITAL.*

By far the greater part of the clinical material in any large hospital is deprived of a measure of its usefulness by the inadequate manner in which clinical notes are made and the lack of method in their collection. No one who has attempted to collect statistical data from the records of a London hospital will readily forget the labour and fatigue involved in searching through its voluminous and often badly written case records to find the disease or symptom groups sought for. In more than one hospital we could name the reports are simply bound up at the end of the year, without any attempt at classification whatever. To enlarge the field of usefulness of the medical history sheet and at the same time to systematise the student's habits of clinical examination the University of California Hospital considered what steps should be taken to render the reports of cases treated readily accessible and at the same time more intelligible to those who wished to investigate a large number of cases of any given disease.

With this end in view they started, three years ago, to develop a complete and admirable though at first sight rather complicated system of indexing and filing, enlisting for its service a trained cataloguer whose whole time is spent in entering the diagnosis cards under their proper headings and elaborating the cross-references. "Causal relationship," says the Report, "and etiology in general have been considered of the greatest importance. We wish to have stated in the diagnosis not only each lesion present, but its connection with other lesions. The diagnosis of bronchitis is not complete unless we know the organism that caused it. The diagnosis of 'chronic endocarditis and syphilis' is not satisfactory unless we are told whether the one was the cause of the other. . . . The same system of double-listing holds for complications. We enter a case under kindey-disease as 'chronic nephritis with coma' and under the nervous system as 'coma due to nephritis,' and in the latter place will also be found 'coma due to diabetes,' 'coma due to brain trauma,' etc."

Cases are not, as with us, recorded by students during their early apprenticeship in the wards, but by the house officers, who have enjoyed two or three years' training in the hospital's methods. A further point and an important one is the standardisation of clinical tests; thus in each ward is kept the "ward reference book," which contains an exact description of various clinical procedures in common use, which have not as yet become well established in text-books. Thus the routine for estimating sugar or urea in the blood or the technique of blood-pressure measurement is exactly laid down, so that anyone reading the reports several years later will be able to judge the value of the clinical record and estimate the probable percentage error.

An elaborate card of large size (21.5 × 28 c.m.) is used for case-taking, and contains spaces for all the observations commonly required for any type of case. By the use of accepted symbols opposite the particular space clerical work is reduced to a minimum, while the card itself serves as a useful guide to the examining physician.

* *Clinical Records in the University of California Hospital.* Reprinted from the *Boston Medical and Surgical Journal,* November 1915.

